# Assessing upper limb function: the Spanish version of the Stroke Upper Limb Capacity Scale (SULCS): cross-cultural adaptation and clinimetric properties

**DOI:** 10.1007/s10072-026-09102-4

**Published:** 2026-05-19

**Authors:** Arantxa Gómez-Ortega, Francesca Lunardini, Natacha León, Jesús Tornero, Raquel Cantero

**Affiliations:** 1 Center for Clinical Neuroscience, University Hospital Los Madroños, M-501, Km 17, 900, 28690 Madrid, Brunete Spain; 2 Universidad de Málaga, Málaga, Spain; 3 Hospital Los Madroños Foundation, Madrid, Spain; 4 Instituto de Investigacion Biomedica, IBIMA Plataforma Bionand), Malaga, Spain

**Keywords:** Stroke, Neurological assessment, Clinimetric properties, Reliability, Validity, Upper limb, Rehabilitation, Functionality, Occupational therapy

## Abstract

**Background:**

More than 70% of stroke survivors suffer from upper extremity paresis, which limits the ability to perform activities of daily living. To guide rehabilitation, specific tools that accurately evaluate upper limb function are essential. The Stroke Upper Limb Capacity Scale (SULCS) is a stroke-specific assessment tool that evaluates functional capacity of the upper limb. It is quick to administer, requires no specialized equipment, and is a cost-effective option for routine clinical use.

**Objective:**

The aims of this study were to adapt the SULCS transculturally to Spanish and to examine its clinimetric properties.

**Methods:**

For cross-cultural adaptation to Spanish, we employed a "translation/back-translation" approach and evaluated its feasibility in routine neurorehabilitation.

A multicenter study was conducted in Spanish post-stroke patients (*n* = 56) to validate the clinimetric properties of the instrument.

**Results:**

The translation into Spanish was completed without major difficulties. Reliability analysis, including test–retest and inter-operator, demonstrated almost perfect agreement for the Spanish SULCS, with weighted Cohen’s kappa values of 0.8755 (95% CI: 0.5991–1.1519) and 0.8988 (95% CI: 0.7343–1.0632) respectively. The minimal detectable change indicated high sensitivity (0.72 at the 95% confidence level). The SULCS showed excellent concurrent validity with the Fugl-Meyer Assessment (*r* = 0.9533), the Action Research Arm Test (*r* = 0.9730), and the Box and Block Test (*r* = 0.9247). No floor or ceiling effects were observed.

**Conclusions:**

The Spanish version of the SULCS presented excellent clinimetric properties. Its characteristics make it a useful tool for assessing upper limb functional capacity in the Spanish post-stroke population.

**Supplementary Information:**

The online version contains supplementary material available at 10.1007/s10072-026-09102-4.

## Introduction

Stroke is a major cause of short-term functional impairment in developed countries [1]. More than 70% of stroke survivors suffer from upper extremity paresis. This condition limits an individual’s ability to perform activities of daily living, as well as social and recreational activities, and therefore requires appropriate care [2]. The fundamental objective of upper limb rehabilitation following a stroke is to optimize the functional capabilities of the paretic arm and hand, facilitating the execution of meaningful activities [3]*.*

Successful rehabilitation should be informed by appropriate assessment of the upper-limb function to objectively evaluate its effectiveness and allow for adjustments to the rehabilitation plan when necessary. To achieve this goal, it is essential to implement standardized and reliable assessment methods that accurately capture upper-limb function, thereby ensuring that rehabilitation strategies are evidence-based and tailored to individual needs.

Although different upper limb function scales are described in the literature [4–7], all of them require sufficient hand function to be performed and are not suitable to assess basic upper limb capacities in patients with poor hand function, as often seen after a stroke. These assessments frequently encounter floor effects and exhibit limited sensitivity in individuals lacking distal function or necessitating specialized equipment [6–8]. Additionally, their time-consuming administration can impede their practical utility [8].

The Stroke Upper Limb Capacity Scale (SULCS) was developed in 2011 at the Sint Maartenskliniek Rehabilitation Centre in the Netherlands to address upper limb function [9], and was the first assessment tool that included items evaluating both basic upper limb functioning (activities that require reduced or no hand functioning) and more demanding upper limb functioning (activities that require intensive distal functioning [9, 10]). This scale was designed to quantify upper limb capacity as a unified construct, encompassing both essential proximal functions and fine distal arm functionalities. Extensive research has demonstrated that the SULCS possesses unidimensional, hierarchical, and internally consistent characteristics, along with robust interrater reliability, construct validity, and responsiveness [9, 11]. Currently, the SULCS is available in Dutch, English, German, Portuguese, French and Italian languages, thereby facilitating its widespread adoption and cross-cultural applicability.

The objectives of this study were i) the translation and cross-cultural adaption of the SULCS to generate a Spanish version, and ii) the validation of its clinimetric properties in post-stroke patients.

## Methods

The study was conducted over a period of 50 months, from March 2019 to May 2023.

### Translation and adaptation of the SULCS to Spanish

Prior to the initiation of the translation and cross-cultural adaptation process, we obtained authorization from the author of the original instrument through email. The study was approved by the Ethics Committee of University of Málaga (CEUMA) (8-2019H).

#### Study design

The adaptation process of the SULCS followed a protocol like the one used in the IQOLA project (International Quality of Life Assessment) (Ware et al., 1998; Bullinger et al., 1998), developed to obtain different versions of the SF-36 questionnaire and the DASH, PRWHE, CTS instruments (Rosales et al., 2002). The methodological process employed was "translation/back-translation".

The original English questionnaire was translated by four bilingual translators with clinical experience whose native language was Spanish. Each translator prepared a translation and assessed the difficulty in obtaining each of the expressions in Spanish, evaluating the conceptual equivalence and clarity of each phrase and response on a scale from zero to one hundred. For the concept of translation difficulty, a score of zero meant that there was no difficulty, and a score of one hundred meant that there was great difficulty in translating the item in question. Referring to the concept of conceptual equivalence, a score of zero rates the worst equivalence and a score of one hundred the best conceptual equivalence in translation. In a first scientific meeting among translators, researchers, and directors, a first consensual Spanish version was obtained, called version 1.0. To measure conceptual equivalence, the initial Spanish version was translated back into English by two bilingual translators residing in Spain whose native language was American English. In a second meeting, the team of researchers and translators compared the two back-translated scales with the original version to identify items or words that were not exactly equivalent. As a result, they reached the consensus version called 2.0 (supplementary material).

**Fig. 1:**
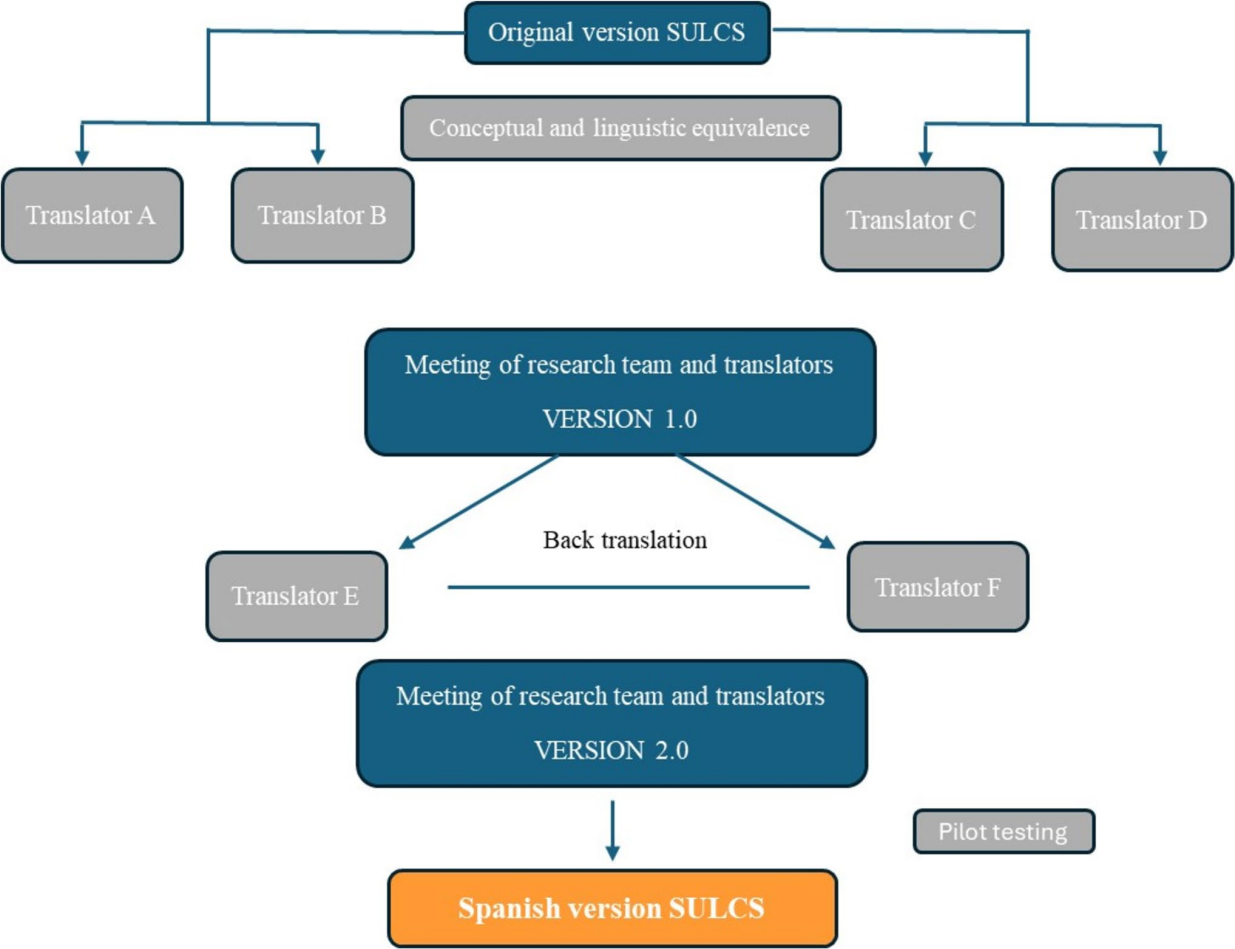
Translation and cross-cultural adaptation of the Stroke Upper Limb Capacity Scale

Version 2.0 was then subjected to pilot testing by a group of three occupational therapists (*n* = 3), who incorporated the scale into their clinical practice with post-stroke patients over a three-week period. Feedback from this pilot confirmed the clarity and applicability of version 2.0, which was therefore adopted as the final Spanish version of the SULCS instrument (Fig. 1).

### Assessment of the clinimetric properties of the Stroke Upper Limb Capacity Scale in Spanish

We evaluated the clinimetric properties of the final Spanish version of the SULCS in patients who had experienced a stroke; specifically, we examined test–retest and inter-rater reliability, measurement error, concurrent validity, and the possible presence of floor or ceiling effects.

Studying all these properties at a time implies a large and time-consuming protocol, where each patient undergoes a fatiguing clinical assessment composed of several scales—some of which repeated multiple times – with the risk of jeopardizing the results. Not to overwhelm the patients with an excessively large protocol, we decided to study such properties in two separate groups of post-stroke patients, one recruited at the Tecan Hand Center (Málaga, Spain) (Group 1), and one at Hospital Los Madroños (Brunete, Madrid, Spain) (Group 2). Prior to the study execution, both institutions obtained the approval by the Ethics Committee of University of Málaga (CEUMA) (8-2019H) and Ethics Committee of Hospital Universitario Severo Ochoa (SULCS_HLM), respectively.

#### Study population

Patients meeting the following criteria were included in the study: age ⩾18 years, hemiparesis resulting from a stroke, and adequate cognitive function to provide consent for study participation and complete study assessments. Moreover, a minimum shoulder function of 1/5, based on the Medical Research Council (MRC) scale [12], was necessary for inclusion.

Of all patients that were eligible and invited, no patient declined to participate. Each patient was given verbal and written information about the study and informed consent was obtained. All procedures performed in this study involving human participants were in accordance with ethical standards. Upon voluntary agreement and completion of the informed consent process, patients were enrolled in the study.

Demographic and clinical data (age, sex, diagnosis, time since stroke and affected side) were obtained through direct interview with the patient.

Sample size calculation was based on previous studies investigating the clinimetric properties of the SULCS [8, 11] that reported high levels of validity (0.85–0.91), inter-rater (0.75–0.93) and test–retest (0.8–0.96) reliability. Given these excellent values, by setting the significance at 5%, we obtained sample size of *n* = 10, *n* = 12, and *n* = 18 patients for the estimation of the validity, the test–retest reliability and the inter-rater reliability, respectively.

#### Study design

The study was an observational study with a cross-sectional design for inter-rater reliability and validity analysis, and a classic cohort design for test–retest reliability.

In addition to the SULCS, patients were assessed with other validated functional scales and tests, administered by trained therapists, to enable comparison across measures. Below we provide a description of the study protocol.

Two independent cohorts were recruited and analyzed separately to avoid excessive patient burden and to minimize fatigue associated with multiple assessments. The data collected from Group 1were used to assess test–retest reliability and validity (Table 1). After signing informed consent, a trained operator first administered the SULCS, followed by the Fugl-Meyer Assessment (FMA), the Action Research Arm Test (ARAT), and the Box and Block Test (BBT). These additional scales were applied in a randomized order to reduce potential bias related to fatigue or other confounding factors, while ensuring that the SULCS was consistently performed first. The SULCS was then re-administered 24 h later by the same operator for the test–retest analysis.

**Table 1:** Patients Demographics

	Group 1	Group 2
Total Number		
#	20	36
Age (years)		
Mean (SD)	69.4 (6.0)	68.8 (11.1)
Sex		
Male	14	24
Female	6	12
Injury Type		
Hemorrhagic	8	14
Ischemic	12	22
Injury side		
Right	15	10
Left	5	26
Time since Stroke (weeks)		
Mean (SD)	3.24 (1.37)	16.3 (6.7)
Protocol		
SULCS test–retest	✓	
SULCS inter-operator		✓
Fugl-Meyer Assessment	✓	✓
Action Research Arm Test	✓	✓
Box and Block Test	✓	✓
Clinical Assessment		
SULCS test/operator 1 (/10)	4.3 (1.1)	4.9(3.4)
SULCS retest/operator 2 (/10)	4.5 (1.1)	5 (3.4)
Fugl-Meyer Assessment (/66)	43.3 (11.4)	40.7 (23.4)
Action Research Arm Test (/57)	21.6 (13.1)	26.3 (23.7)
Box and Block Test (/150)	9.2 (10.1)	14 (16.4)

Demographic and clinical characteristics of patients in Group 1 and Group 2. The distribution of stroke type, side of lesion, and assessment protocols are shown for each group. Clinical assessment scores are reported as mean and standard deviation

For the evaluation of inter-rater reliability and validity, the study was conducted with Group 2. Assessments took place in two phases on the same day. In the first phase, and after providing informed consent, the SULCS was administered by the first trained operator. After a minimum interval of one hour, the second phase was conducted, during which a second trained operator again administered the SULCS. The order in which the two operators carried out the assessments was randomized to minimize potential rater-related bias. Immediately following the second SULCS assessment, the FMA, ARAT, and BBT were administered, also in a randomized order, following the same methodological approach described for the first cohort to limit the influence of fatigue and other confounders, and maintaining the SULCS as the initial assessment.

The SULCS comprises ten items representing various daily activities of increasing difficulty. Items 1–3 involve tasks focusing on the proximal upper limb capacity without requiring active wrist and finger movements. Items 4–7 necessitate basic control of wrist and finger movements, while items 8–10 demand advanced control to demonstrate higher levels of dexterity. The scoring system is dichotomous, with a score of 0 assigned if the patient is unable to perform the task as described, and a score of 1 if the task is executed correctly. The minimum and maximum sum scores range from 0 to 10, respectively. Implementing a "start and stop" rule can streamline administration time. In our study, we initiated with the easiest task and concluded when the patient failed to perform three consecutive tasks [9].

The ARAT is a 19-item observational measure used to assess upper extremity performance (coordination, dexterity and functioning) in stroke recovery, brain injury and multiple sclerosis populations. The ARAT scores range from 0 to 57 points, with a maximum score of 57 indicating optimal performance. Scores of less than 10 points, between 10 and 56 points, and 57 points are associated with poor, moderate, and good recovery, respectively [13]. There is moderate to strong evidence supporting the intra-rater reliability and responsiveness of the test [14].

The original FMA [15] was developed to evaluate the functional status of stroke patients and consists of six domains: upper extremity motor function, lower extremity motor function, balance, sensation, range of motion, and joint pain, comprising a total of 113 items and 226 points. This scale has been validated in Spanish [16] with a test–retest reliability ICC of 0.987 for the upper limb and a Cronbach’s coefficient of 0.98.

The BBT [17, 18] is one of the most frequently utilized evaluations for assessing gross manual dexterity within the domain of mobility (ICF domain d4). Renowned for its simplicity and efficiency, the BBT offers a straightforward assessment that can be swiftly completed, making it particularly suitable for individuals with restricted hand function. Beyond its primary focus on gross dexterity, the BBT also evaluates other crucial motor components, including eye-hand coordination and the ability to navigate partition walls [19]. The BBT involves the transfer of wooden blocks measuring 2.5 cm × 2.5 cm × 2.5 cm from one compartment of a box to another within a duration of 1 min. The normative data collected on the BBT are consistent with other tests of manual dexterity [20].

### Statistical analysis

All statistical analyses were performed using MATLAB R2023b (MathWorks, Natick, MA, USA). The significance level was set at 5%.

#### Reliability

Given the discrete nature of the clinical scoring, reliability of the Spanish version of the SULCS was assessed using the Weighted Cohen’s Kappa [21].

For interpretation of the Weighted Cohen’s Kappa, values ≤ 0 indicate no agreement; between 0.01–0.20 indicate none to slight agreement; between 0.21–0.40 indicate fair agreement; between 0.41–0.60 indicate moderate agreement; between 0.61–0.80 indicate substantial agreement; between 0.81–1.00 indicate almost perfect agreement [22].

#### Measurement error

The standard error of measurement was calculated to determine the precision of individual scores, and the minimal detectable change was computed as a measure of responsiveness.

#### Concurrent validity

The concurrent validity of the SULCS was evaluated by calculating Spearman’s correlations between SULCS scores and reference scales (FMA, ARAT, and BBT). Correlations were interpreted as follows: weak (< 0.4), moderate (0.4–0.69), or strong (≥ 0.7).

#### Floor and ceiling effects

Floor and ceiling effects were calculated as the proportion of patients with the minimum and maxim total score. Since a minimal shoulder function of 1 (based on the MRC scale) was requested for inclusion, the minimum score was not set to 0, but to 10.0% of the maximum score, which resulted in a minimum score of 1 for the SULCS. We considered a floor or ceiling effect to be present, when the above proportion overcame the threshold of 15%, as suggested by Terwee and colleagues [23].

## Results

### Translation and adaptation of the SULCS to Spanish

The translation process from the original version to the Spanish version was completed without major issues. During the back-translation phase, minor discrepancies were identified and addressed concerning the phrasing of item 2 (Holding an object between the trunk and upper limb). Specifically, the question arose as to whether the item should specify if the elbow should remain extended or allow for flexion. Following a meeting with the research team, the decision was made to maintain the same activity structure as in the original version to preserve consistency.

Pilot testing of the Spanish version (Version 2.0) did not reveal any significant issues related to comprehension or application, supporting its usability in this context. No uncertainty regarding item administration was found. The Spanish version (Version 2.0) of the SULCS is available in the Supplementary Material.

### Assessment of SULCS measurement properties

A total of 56 participants who had experienced a stroke in the past 6 months participated in the study:20 patients from the Tecan Hand Center (Málaga, Spain) (Group 1), and 36 patients from Hospital Los Madroños (Brunete, Madrid, Spain) (Group 2) (Table 1) No statistical between-group differences emerged in terms of age, sex, injury type, and scores obtained from clinical assessment (SULCS test/operator 1; SULCS test/operator 2; ARAT, FMA, BBT). The two groups slightly differed in terms of injury side and time since stroke, although all participants were acute and subacute patients. There was no loss to follow-up in the test–retest assessment.

#### Test–retest reliability

The test–retest results for the SULCS were assessed as part of the evaluation of its adaptation and clinimetric properties in Group 1, composed of a total of 20 patients.

**Fig. 2:**
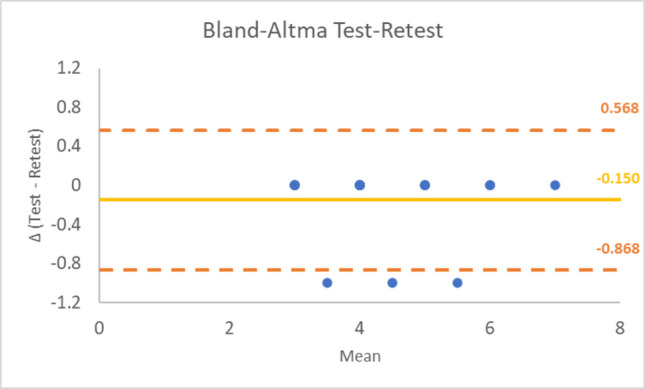
Bland–Altman plot for the test–retest reliability of the Spanish version of the SULCS. Each point represents the difference between the two measurements plotted against their mean. The mean difference of − 0.150 indicates minimal systematic bias between test and retest. The random and horizontally oriented distribution of the points suggests the absence of proportional or systematic bias across different levels of patient ability. Most observations fall within the limits of agreement (− 0.868 to 0.568), supporting the stability of the measure across repeated administrations

The results from the test–retest analysis indicated an almost perfect agreement for the Spanish version of the SULCS, with a weighted Cohen’s Kappa of 0.8755 (95%-confidence interval: 0.5991–1.1519). This suggests that the SULCS is highly consistent when administered multiple times to the same individuals, reinforcing its reliability as a clinimetric tool for assessing upper limb function. The Bland–Altman plot (Fig. 2) shows good agreement, with differences mostly assuming a value of 0.

#### Inter-operator reliability

**Fig. 3:**
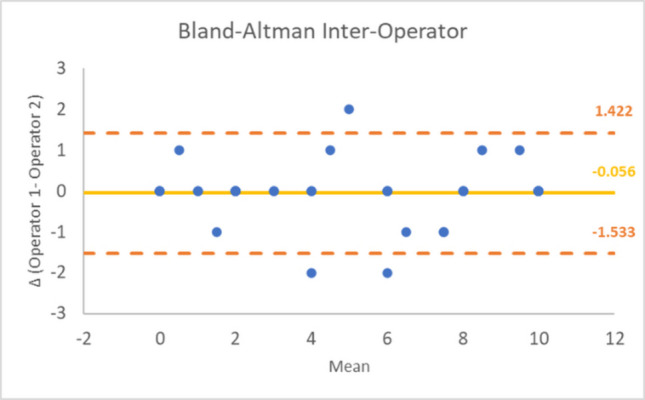
Bland–Altman plot for the inter-operator reliability of the Spanish version of the SULCS. Each point represents the difference between the evaluations performed by the two operators plotted against their mean. The mean differences was −0.056, indicating minimal bias between the assessments of operator 1 and operator 2. The random and horizontal distribution of the points suggests the absence of bias in patients with different levels of functional ability. Most observations fall within the limits of agreement (−1.533 to 1.422), supporting a good level of agreement between operators

Inter-operator reliability for the SULCS was assessed as part of the evaluation of its adaptation and clinimetric properties in Group 2, composed of a total of 36 patients. The analysis reached an almost perfect agreement, with a Weighted Cohen’s Kappa of 0.8988 (95%-confidence interval: 0.7343–1.0632). The Bland–Altman plot (Fig. 3) shows good agreement, with almost all the point falling between the limits of agreement.

#### Standard error of measurement

The standard error of measurement was calculated to be 0.26, indicating a small degree of error in individual scores and suggesting a high level of precision in the instrument. The minimal detectable change was 0.72 at the 95% confidence level. This means that any change greater than 0.72 points in a patient’s score can be considered a significant and real change in their upper limb capacity.

The low standard error of measurement value demonstrates that the Spanish version of the SULCS provides precise measurements with minimal variability. The minimal detectable change value establishes a benchmark for interpreting changes in scores; for example, if a patient’s score increases by at least 0.72 points following an intervention, this improvement can be confidently attributed to actual progress rather than measurement error.

#### Concurrent validity

The validity of the SULCS was computed by correlating the scores obtained in the SULCS and in the other functionality scales for the pooled dataset of patients (Group 1 and Group 2 together). All correlations were statistically significant (*p* < 0.001). The SULCS shows strongly positive correlations with the FMA, ARAT, and BBT, supporting the concurrent validity of the scale (Table 2).

**Table 2:** Correlations with reference clinical scales

Reference Scale	Correlation Coefficient (Rho)	*p*-value
FMA	0.9533	< 0.001
ARAT	0.9730	< 0.001
BBT	0.9247	< 0.001

Concurrent validity of the Spanish version of the SULCS with established upper-limb outcome measures

#### Floor and ceiling effects

No floor, nor ceiling effects emerged for the Spanish version of the SULCS in the pooled dataset of patients (Group 1 and Group 2 together). Indeed, only 4 (7.1%) and 6 (10.7%) out of the 56 patients reported the lowest (1) and highest (10) scores, respectively. Both proportions are below the threshold of 15%.

## Discussion

In this study, the translation and cross-cultural adaptation of the SULCS into Spanish were carried out, along with the evaluation of several clinimetric properties, including test–retest and inter-rater reliability, measurement error, the concurrent validity of the scale in comparison with other instruments of the same category, and the evaluation of possible floor and ceiling effects.

We successfully achieved cross-cultural adaptation of the SULCS into Spanish, showing that it can be easily applied in the clinical context, without any obstacle in its adoption.

The scale showed excellent reliability, with perfect agreement in both test–retest and inter-rater assessments. In terms of validity, the Spanish SULCS demonstrated strong correlations with the FMA, ARAT, and BBT. These findings are consistent with those obtained in previous adaptations to other languages, such as Dutch [11], Brazilian [24], Portuguese [25], German [8], and Italian [26], thereby confirming the excellent clinimetric properties of the scale.

As for test–retest reliability, it is worth pointing out that, although the 24-h interval between assessments -adopted also in previous studies [8, 26]- minimizes the likelihood of genuine clinical change, it may not fully eliminate the possibility of memory of familiarity effects, which may slightly influence the test–retest reliability estimates.

This scale may serve as a highly useful tool in clinical practice due to its ease and speed of administration (approximately six minutes), following the *start & stop* rules [9], compared to other commonly used scales in clinical settings, such as the FMA, which requires around 30 min for administration [27].

Additionally, the SULCS stands out for its low cost, as the required materials consist of everyday objects. The use of these familiar items facilitates patient comprehension and test execution, making it suitable even for individuals with language difficulties who retain a certain level of understanding. On the other hand, other standardized assessments, like the ARAT, provide valuable data on functional capacity, but their clinical applicability may be limited due to the high cost.

Another strength of the SULCS is its classification into groups of three items, encompassing tasks requiring no manual ability, poor manual ability, and advanced manual capacity. This structure enables its application across a broad spectrum of patients. In contrast, the BBT and the ARAT can only be administered to individuals with sufficient manipulative ability to move objects, thereby limiting their use in clinical practice.

This study has some limitations that should be acknowledged. A deliberately heterogeneous sample of acute and subacute patients was included, encompassing a wide range of ages, levels of manipulative ability, and diverse types of stroke (hemorrhagic, ischemic). This diversity is crucial to ensure that our findings are generalizable to the broader target population. However, it should be acknowledged that the two recruited groups—Group 1 (test–retest reliability) and Group 2 (inter-rater reliability)—slightly differed in terms of time since stroke and side of lesion. Although all participants were in the acute or subacute phase, and no significant between-group differences were observed in functional scale scores, this heterogeneity may represent a potential source of variability and should be considered when interpreting the results.

In addition, no formal neuropsychological assessment was performed, although the inclusion criteria required patients to have sufficient ability to understand instructions and follow commands to complete the assessment. Consistent with the side of cerebral involvement, some patients with left-hemisphere lesions presented mild language difficulties, and some with right-hemisphere lesions showed mild hemineglect-like symptoms. While these deficits did not interfere with test administration, they may have influenced performance, reflecting individual functional ability rather than affecting the clinimetric properties of the scale.

Finally, muscle tone was not recorded as a specific variable, as the SULCS is designed to assess upper limb functional capacity independently of tone. The progressive structure of the scale (from proximal basic tasks to activities requiring advanced hand control) allows its application in patients with different levels of muscle tone, with any influence of tone inherently reflected in the final score.

It is important to highlight that, during the validation process, a significant variability in motor capacities was observed among subjects who obtained the same score in all the upper limb functionality scales (SULCS, ARAT, FMA, BBT). This limitation, common to all clinical scales with discrete scores and low granularity, restricts the ability to adequately differentiate patients’ severity. Future research should address this issue to improve the longitudinal monitoring of functional changes and, consequently, optimize the rehabilitation plan.

## Conclusion

The translation process from the original version into Spanish was completed successfully, without encountering any substantial linguistic or conceptual difficulties.

The Spanish version of the SULCS exhibited excellent clinimetric properties. High test–retest reliability (weighted Cohen’s Kappa = 0.8755) and perfect agreement in inter-operator (weighted Cohen’s Kappa = 0.8988) confirmed the robustness of the instrument. Strong significant correlations (rho > 0.92) with reference scales supported the SULCS concurrent validity. The measurement error analysis demonstrated high precision and sensitivity to change, establishing the SULCS as a reliable tool for clinical and research use in assessing upper limb capacity. Finally, nor floor neither ceiling effects were observed.

The excellent clinimetric properties, the ease and speed of administration, and the cost-saving make this scale a useful tool for assessing the functional capacity of the paretic limb in the Spanish population.

## Supplementary Information

Below is the link to the electronic supplementary material.

Supplementary file1 (PDF 82 KB)

Supplementary file2 (PDF 705 KB)

## Data Availability

The data that support the findings of this study are available from HLM/Málaga but restrictions apply to the availability of these data, which were used under license for the current study, and so are not publicly available. Data is, however, available from the authors upon reasonable request and with permission of HLM.
