# Supplementary material for: Assessing upper limb function: the Spanish version of the Stroke Upper Limb Capacity Scale (SULCS): cross-cultural adaptation and clinimetric properties
Source: Neurol Sci. 2026 May 19;47(6):498. doi: 10.1007/s10072-026-09102-4 (PMC13183723; doi:10.1007/s10072-026-09102-4)
Supplement: Supplementary file 1 — Supplementary file1 (PDF 82 KB) [file 10072_2026_9102_MOESM1_ESM.pdf]

APÉNDICE A: INSTRUCCIONES GENERALES Y LISTA DE MATERIALES PARA SULCS

Instrucciones y explicación

1. Las 10 tareas de la lista están en orden de dificultad y complejidad.
2. Las tareas se realizan de pie o sentado. Está permitido desviarse del orden prescrito de las tareas por razones prácticas.
3. Todas las tareas deben realizarse sin ayuda.
4. Es importante calificar si la tarea se puede realizar de acuerdo con las instrucciones (capaz/no se puede), no la calidad de cómo se realiza.
5. Si es necesario, está permitido repetir las instrucciones o demostrar la tarea.
6. Se puede decidir comenzar con la tarea 1 o la tarea 10 evaluando de antemano el nivel de capacidad de las extremidades superiores. Comience con la tarea 1 para baja capacidad y la tarea 10 para alta capacidad.

Materiales Test.

- Una mesa de altura ajustable
- Una silla
- Un bolígrafo
- Una revista semanal de aproximadamente tamaño A4 o Carta (± 210 gramos/7 ½ onzas) doblada por la mitad a lo largo.
- Un paño de cocina
- Un frasco vacío ± 400 gramos, con una tapa de plástico de rosca (± 20 mm de alto, diámetro de la tapa ± 77 mm). La tapa cerrada y el bote se marcan con un rotulador para que cada vez que se vuelva a cerrar el bote, como sucedería después de un uso normal, las marcas se alineen (objetivo: asegurar que el grado de dificultad sea el mismo cada vez). se realiza la prueba).
- Un vaso largo (diámetro ± 55 mm ± 150 mm de alto)
- Una pelota de tenis
- Un peine
- Una camisa de hombre
- Un cronómetro
- Tres monedas de diferentes tamaños: una moneda de 50 céntimos de euro (diámetro ± 23 mm), una moneda de 2 céntimos de euro (diámetro ± 17 mm) y una moneda de 1 céntimo de euro (diámetro ± 15 mm), o sus equivalentes en tamaño y peso
- Apéndice B

Información del contacto
Puede encontrar más información sobre el contenido de SULCS en línea en:
http://www.maartenskliniek.nl/kenniscentrum-rde/innovaties

APÉNDICE B: TAREAS 9 Y 10

TAREA 9
Nota: Esta tarea se realiza solo si el lado afecto es el dominante

Escribe tu nombre entre las dos líneas sin sobrepasarlas.

Nota: Esta tarea se realiza solo si el lado afecto es el no dominante

Dibuja tres círculos entre los círculos impresos, sin tocar los bordes de los círculos impresos o cualquier círculo ya dibujado:

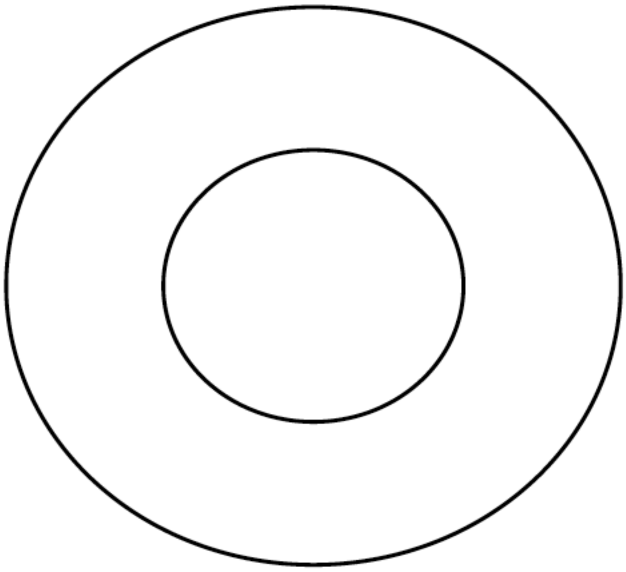

TAREA 10

Coloca las monedas en la posición correcta

50 euro cent **2 euro cent** **1 euro cent**
