# Supplementary material for: Assessing upper limb function: the Spanish version of the Stroke Upper Limb Capacity Scale (SULCS): cross-cultural adaptation and clinimetric properties
Source: Neurol Sci. 2026 May 19;47(6):498. doi: 10.1007/s10072-026-09102-4 (PMC13183723; doi:10.1007/s10072-026-09102-4)
Supplement: Supplementary file 2 — Supplementary file2 (PDF 705 KB) [file 10072_2026_9102_MOESM2_ESM.pdf]

SULC ESPAÑOL. Escala de capacidad del miembro superior en el ICTUS.

Nombre del paciente:
Año de nacimiento:
Diagnóstico:
Fecha del ICTUS:
Nombre del terapeuta:
Fecha:

Apéndice A: Instrucciones generales y lista de material de prueba para SULCS
Apéndice B: Tareas del cuestionario SULCS 9 y 10.

Puntuación
0 = el paciente no puede realizar la tarea de la manera descrita
1 = el paciente es capaz de realizar la tarea de la manera descrita

Nota
Opción 1: comience la prueba en la tarea 1 y avance desde allí. Cuando no se pueden realizar 3 tareas consecutivas, la prueba puede detenerse. Cada elemento restante se puntúa con 0.
Opción 2: comience la prueba en la tarea 10 y retroceda desde allí. Cuando se pueden realizar 3 tareas consecutivas, la prueba se puede detener. Cada ítem restante se puntúa con 1.

	Descripción	Imagen	Puntuación
**Qué**	Usar el antebrazo como apoyo durante la sedestación	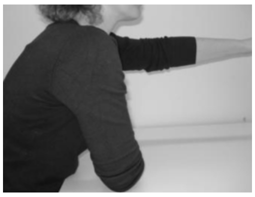	
**Cómo**	Estirándose hacia delante apoyándose en el antebrazo afecto		
Preparación	El paciente está sentado delante de una mesa. El antebrazo afectado apoyado en el mesa, paralelo al borde donde se sienta el paciente. Se coloca un bolígrafo sobre la mesa, frente al codo afectado y lo suficientemente lejos como para que sea necesario el movimiento del tronco para alcanzar el bolígrafo.		
Tarea	El paciente alcanza a recoger el bolígrafo con el brazo sano. El antebrazo afecto se utiliza como apoyo.		
Nota	El brazo afecto no debe moverse cuando el paciente se estira hacia adelante para recoger el bolígrafo.		
**Qué**	Sujetar un objeto entre el tronco y el miembro superior	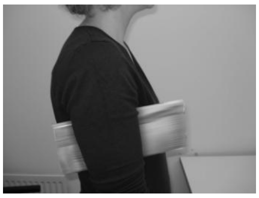	
**Cómo**	Mantener el brazo afecto presionado contra el lateral del tronco		
Preparación	El paciente está de pie (sentado, si es necesario) delante de una mesa. Una revista doblada por la mitad a lo largo está sobre la mesa. El brazo afecto se mantiene colgando libremente pegado al cuerpo.		
Tarea	El paciente coge la revista con la mano no afecta y la coloca entre el torso y la parte superior del brazo afectado.		
Nota	La revista debe sujetarse firmemente durante 10 segundos. El terapeuta lo comprueba si es necesario, tirando ligeramente de la revista.		
**Qué**	Deslizar un objeto sobre la mesa mientras permanece sentado	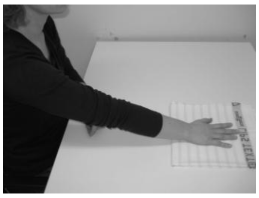	
**Cómo**	Controlando el movimiento de deslizamiento del brazo afecto		
Preparación	El paciente está sentado delante de una mesa. La mano afectada está sobre la mesa sobre un paño de cocina doblado en cuatro, con la palma hacia abajo y los dedos apuntando hacia adelante.		
Tarea	El paciente empuja el paño de cocina hacia adelante sobre la mesa.		
Nota	El codo debe extenderse al menos 160° y puede levantarse de la mesa. No es necesario que los dedos estén completamente extendidos.		
**Qué**	(Parcialmente) Desenroscar una tapa.	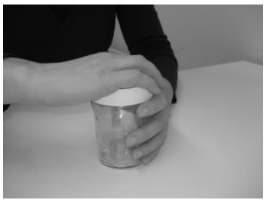	
Preparación	El paciente está sentado delante de una mesa con ambos brazos sobre la mesa. Un tarro cerrado con una tapa de rosca de plástico de 15 cm se coloca frente al paciente en la mesa.		
Tarea	El paciente sostiene el frasco firmemente sobre la mesa con la mano no afecta, utilizando la mano afectada, gira la tapa al menos un cuarto de vuelta.		
Nota	El frasco debe permanecer en el mismo lugar sobre la mesa y no puede girar.		
**Qué**	Coger un vaso de agua y beber	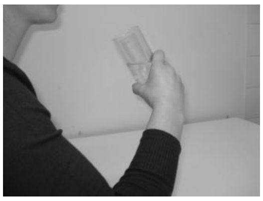	
Preparación	El paciente está sentado delante de una mesa con ambos brazos sobre la mesa. Se coloca un vaso lleno a la mitad de 15cm de diámetro en la mesa frente al paciente.		
Tarea	El paciente coge el vaso de agua de la mesa con la mano afecta, bebe un trago y vuelve a poner el vaso en la mesa sin derramar el líquido.		
Nota	La mano no afecta no se usa		

**Qué**	**Agarrar una pelota presentada desde un ángulo alto**
Preparación	El paciente está de pie (sentado, si es necesario) sin ningún otro apoyo a su alcance. El terapeuta sostiene una pelota de tenis por delante y por encima del hombro afectado de tal manera que el paciente tiene que extender completamente el brazo afectado y debe levantar el brazo ±120° para agarrar la pelota de tenis.
Tarea	El paciente alcanza la pelota y la toma con la mano afectada.

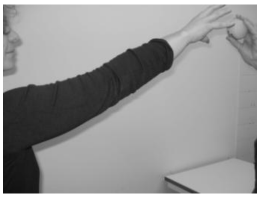

**Qué**	**Peinarse el cabello**
Preparación	El paciente está de pie (sentado, si es necesario) delante de una mesa. Un peine está al alcance de la mano sobre la mesa.
Tarea	El paciente se peina con al menos dos pasadas en la parte superior y a cada lado de la cabeza.
Nota	La cabeza debe mantenerse recta. El paciente debe llegar a los lados desde arriba o desde los lados. Cuando la situación personal sea menos adecuada para esta prueba, se debe hacer un movimiento de "como si".

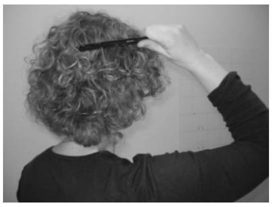

**Qué**	**Abrochar botones**
**Cómo**	**Trabajando con las dos manos**
Preparación	El paciente está sentado frente a una mesa. Una camisa de caballero está en la mesa frente al paciente. El cuello está en la parte superior, mirando hacia arriba. El botón superior está abrochado, todos los demás están desabrochados.
Tarea	El paciente abrocha cuatro botones en 60 segundos con ambas manos.
Nota	Los dedos afectados deben usarse activamente, ya sea para sostener el material o el botón, o para mantener abierto el ojal.

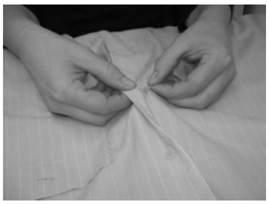

**Qué**	**Escribir**
**Cómo**	**Ver apéndice B**
Preparación	El paciente está sentado delante de una mesa. La hoja de papel, incluida como Apéndice B, está a 15 cm frente al paciente sobre la mesa. Un bolígrafo está en la hoja de papel.
Tarea	(Versión 1: el lado afectado no es el lado dominante)<br>El paciente coge el bolígrafo y dibuja tres círculos entre los dos círculos de la hoja, sin tocar los bordes de los círculos impresos ni de ningún círculo ya dibujado.
Nota	Explique la instrucción en su totalidad de antemano y sugiera que el paciente comience cerca del círculo interno. El paciente puede mover la hoja de papel.
Tarea	(Versión 2: el lado afectado es el lado dominante)<br>El paciente coge el bolígrafo y escribe legiblemente su nombre y apellido con su propia letra entre líneas.
Nota	El paciente puede mover la hoja de papel.

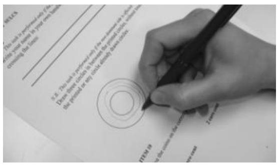

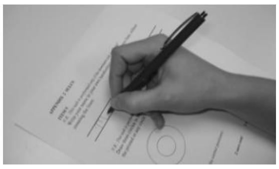

**Qué**	**Manipular monedas**
**Cómo**	**Ver apéndice B**
Preparación	El paciente está sentado delante de una mesa. La hoja de papel, incluida como Apéndice B, está sobre la mesa, directamente frente al paciente. Hay una moneda de 50 céntimos de euro, una moneda de 2 céntimos de euro y una moneda de 1 céntimo de euro (o sus equivalentes en tamaño y peso) sobre la mesa. El antebrazo afecto está sobre la mesa con la mano hacia arriba. Usando la mano no afectada, el paciente coloca las monedas en la mano afectada.
Tarea	El paciente manipula las monedas dentro de la mano afectada, una a la vez entre las puntas de los dedos pulgar e índice y las coloca en sus posiciones designadas en la hoja.
Nota	No importa en qué orden se coloquen las monedas en sus lugares designados. Durante la manipulación, el antebrazo debe descansar sobre la mesa.

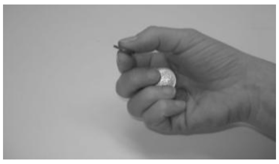

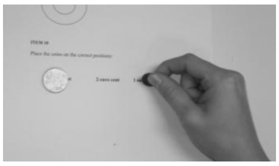

PUNTUACIÓN TOTAL SULCS
